# From Diagnostic Challenge to Clinical Success: Rapid Multiplex PCR Identification of Cryptosporidium in HIV‐Associated Refractory Diarrhea: A Case Report

**DOI:** 10.1002/ccr3.72301

**Published:** 2026-03-13

**Authors:** Yudai Kono, Mayu Arase, Kohei Ukai, Keiko Soneda, Shinya Yamamoto, Kazuhiko Ikeuchi, Eisuke Adachi, Yusuke Nomura, Yoshimi Higurashi, Shinji Izumiyama, Kazuki Honjo, Yu Miyakawa, Sozaburo Ihara, Hiroyuki Abe, Mitsuhiro Fujishiro, Shu Okugawa, Takeya Tsutsumi

**Affiliations:** ^1^ Department of Infectious Diseases The University of Tokyo Hospital Tokyo Japan; ^2^ Department of Infectious Diseases and Applied Immunology, IMSUT Hospital The Institute of Medical Science, the University of Tokyo Tokyo Japan; ^3^ Department of Infection Control and Prevention The University of Tokyo Hospital Tokyo Japan; ^4^ Department of Parasitology National Institute of Infectious Diseases Tokyo Japan; ^5^ Department of Gastroenterology The University of Tokyo Hospital Tokyo Japan; ^6^ Department of Pathology The University of Tokyo Hospital Tokyo Japan

**Keywords:** *cryptosporidium*, gram stain, HIV, multiplex PCR

## Abstract

Cryptosporidiosis remains a challenge in people with HIV despite its decreased incidence in developed countries. We report a case of severe diarrhea in a person with HIV where Gram stain findings prioritized cryptosporidium in the differential diagnosis. Multiplex PCR enabled early diagnosis and treatment, highlighting its value in clinical management.

## 1 Introduction

*Cryptosporidium* is a protozoan parasite that causes cryptosporidiosis, a diarrheal disease affecting humans and animals. *Cryptosporidium* species infect the gastrointestinal tract, leading to symptoms such as watery diarrhea, abdominal pain, nausea, and vomiting. The infection is transmitted through the ingestion of oocysts that are excreted in the feces of infected hosts and can contaminate water sources and food [1]. Sexual transmission of *Cryptosporidium* also occurs, especially among sexually active men. *Cryptosporidium* causes approximately 20% of all pediatric cases of diarrhea in developing countries and causes fatal complications in people with human immunodeficiency virus (HIV) [2]. *Cryptosporidium* is a pathogen that causes severe diarrhea in patients with acute immunodeficiency syndrome (AIDS), and early diagnosis and treatment can significantly affect prognosis [3]. *Cryptosporidium* is responsible for > 8 million foodborne illness cases worldwide annually [4]. However, in developed countries in which environmental contamination rates are low and strong antiretroviral therapy (ART) is widely available, the incidence of cryptosporidiosis among individuals with HIV has declined. For instance, the incidence of cryptosporidiosis in individuals with HIV is currently < 1 case per 1000 person‐years in the United States [5].

Conventional methods for the rapid diagnosis of cryptosporidiosis are difficult. The lack of sensitivity of stool microscopy and polymerase chain reaction (PCR) tests, which target some specific pathogens and are subject for outsourcing, leads to delayed diagnosis. Contrastingly, a multiplex PCR such as the film array gastrointestinal panel is increasingly used to identify the causative microorganisms of diarrhea.

Here, we present a case of refractory diarrhea that was successfully and promptly diagnosed as cryptosporidiosis using the multiplex PCR, enabling effective therapeutic intervention before obtaining pathological findings from upper and lower gastrointestinal endoscopies.

## 2 Case History and Examination

A 48‐year‐old man presented to a previous hospital with diarrhea, weight loss of 25 kg over a month, and persistent vomiting for the past two months. He was initially treated with symptomatic therapy but experienced a recurrence of nausea, along with a new onset of cough, sputum production, and dyspnea. As dyspnea worsened, he was admitted to a previous hospital one month prior.

The patient's medical history included childhood asthma and syphilis. No HIV testing was performed for more than a decade. The toxoplasma antibody was positive. He did not drink alcohol, smoked approximately 20 cigarettes daily, and never traveled abroad. There was no significant family medical history. He was diagnosed with pneumocystis pneumonia (PCP) at the previous hospital and treated with trimethoprim–sulfamethoxazole (TMP‐SMX) therapy (15 mg/kg/day) with adjunctive prednisone (80 mg/day). Prednisone therapy was tapered and discontinued. After the PCP was confirmed, HIV infection was identified with a CD4+ count of 36/μL and HIV‐RNA level of 10,000 copies. He had been living with a male partner for > 10 years and had sexual intercourse with multiple men three months previously. He was transferred to our hospital because of difficulty in diagnosis and treatment for diarrhea.

Upon hospital transfer, omeprazole and loperamide were administered. Upon examination, he was afebrile with a temperature of 37.2°C, blood pressure of 110/60 mmHg, pulse of 110/min, respiratory rate of 16/min, and an oxygen saturation of 97% on room air. His height was 176.6 cm and weight was 67.6 kg. There was no evidence of oral thrush. Cardiovascular examination findings were normal, lung auscultation was clear, and abdominal examination showed no hepatosplenomegaly. A rash with pruritic erythematous papules was observed on the inner thighs, axillae, and buttocks. Blood tests revealed platelet was 2, 6 × 10^4^/μL. Because of the thrombocytopenia and rash, TMP‐SMX was discontinued upon admission and atovaquone was initiated after four days. The platelet improved to 19 × 10^4^/μL, suggesting drug‐induced thrombocytopenia.

## 3 Differential Diagnosis, Investigations, and Treatment

Initial differential diagnosis of chronic diarrhea included CMV enteritis, amebiasis, disseminated MAC infection, inflammatory bowel disease, drug‐induced diarrhea, and protozoan infection (*Cryptosporidium*, *Cyclospora*, *Giardia*).

**FIGURE 1:**
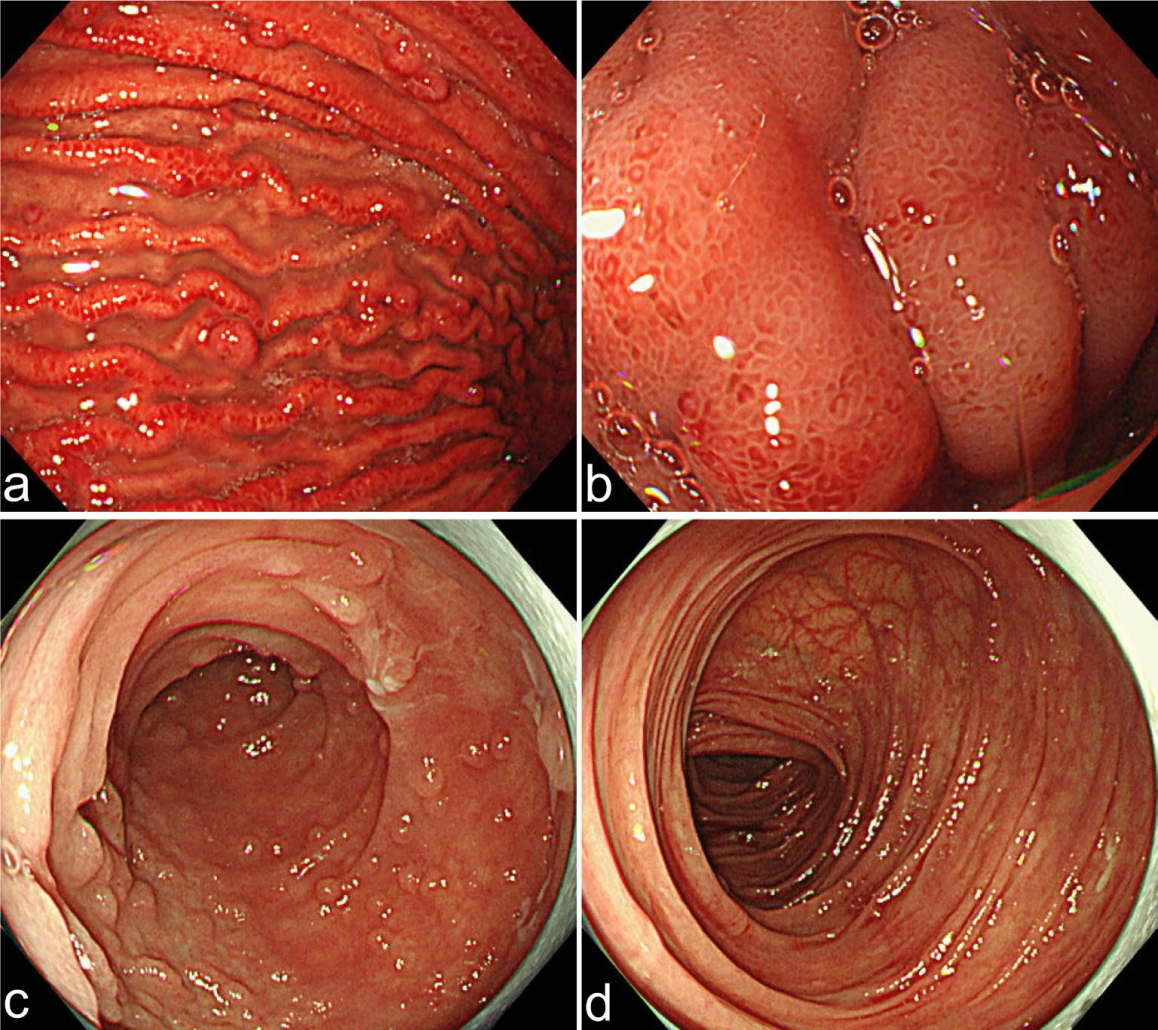
Representative images of endoscopic findings within (a) the gastric body, (b) the anterior wall of the duodenal bulb, (c) the terminal ileum, and (a) the transverse colon.

**FIGURE 2:**
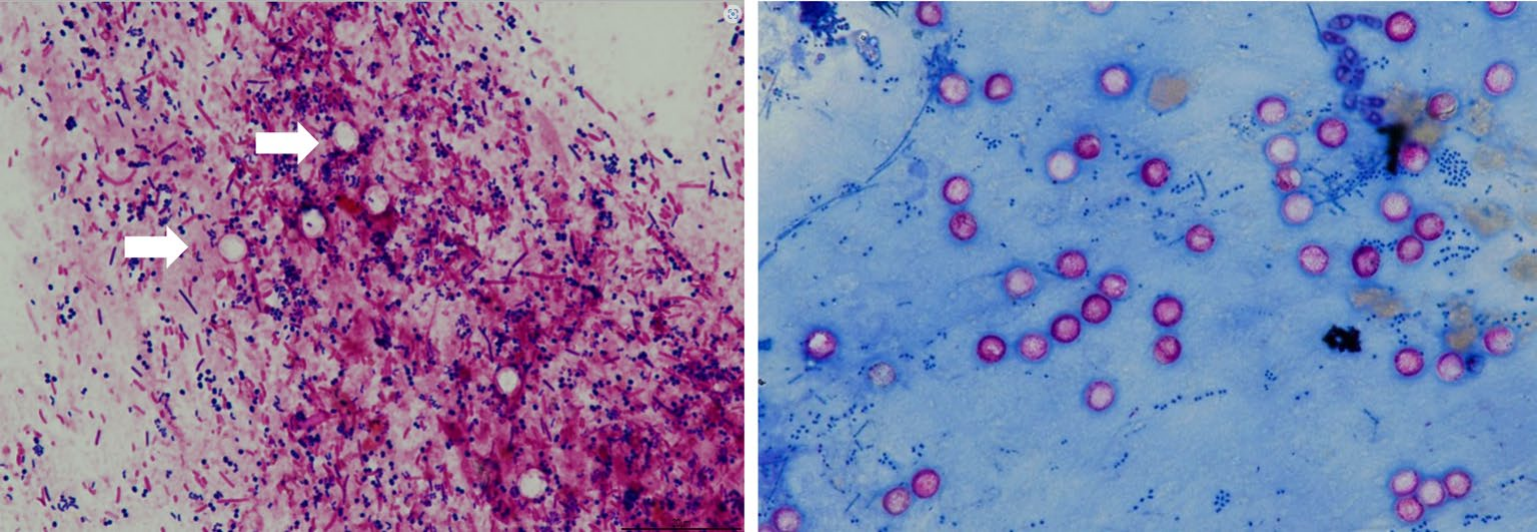
Gram stain and acid‐fast staining of the stool sample reveals structures with a white halo.

**FIGURE 3:**
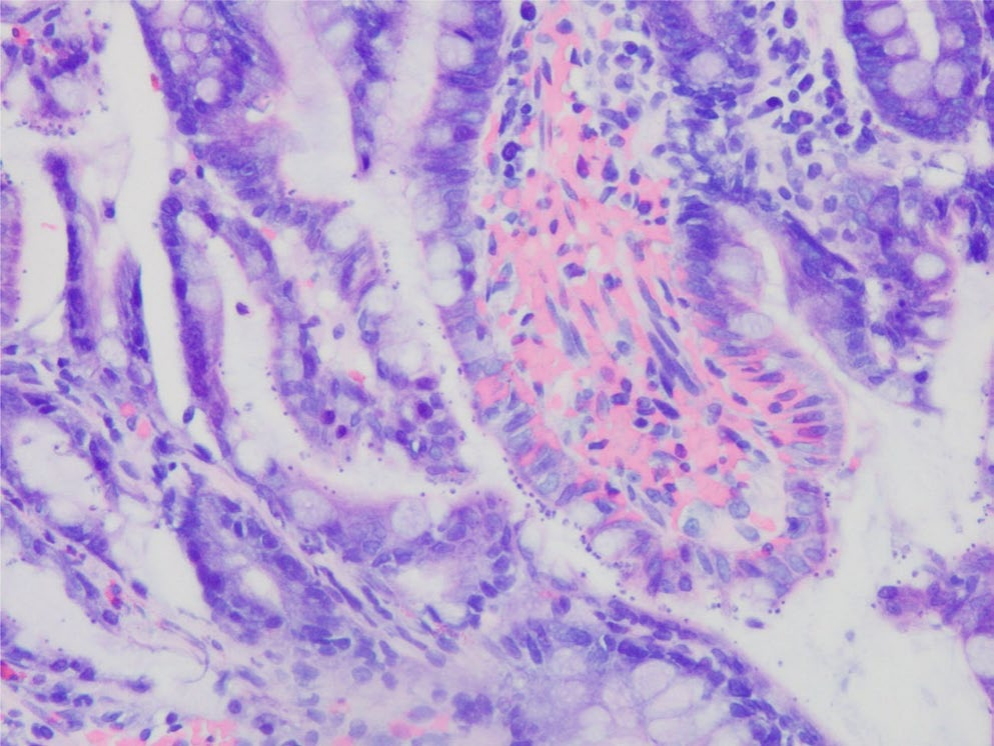
Giemsa staining of the ileum with numerous round parasites attached to the mucosal cells.

Stool culture showed no evidence of enteritis‐causing bacteria, including *Clostridioides difficile*, or protozoa, such as *Entamoeba histolytica* . The stool amoebic antigen ( *E. histolytica* QUIK CHEK, Techlab, Blacksburg, VA, USA) was negative. Contrast‐enhanced computed tomography revealed no significant findings. The upper gastrointestinal endoscopy revealed diffuse erythema and edematous changes mainly in the gastric body, with focal areas of hyperplastic changes, and marked edematous changes in the duodenum. A colonoscopy revealed no remarkable findings (Figure 1). However, biopsies were taken from the stomach, the terminal ileum, and colon to collect histopathological information. Eight days after admission, bictegravir/emtricitabine/tenofovir alafenamide (BIC/FTC/TAF) was initiated. A Gram stain of the repeat stool specimen revealed structures with a white halo (Figure 2), suggestive of a protozoal infection (e.g., *Cryptosporidium*, *Cyclospora*). Subsequently, we added Giemsa staining of the stool and terminal ileum, which demonstrated numerous round shaped parasites were attached to the mucosal cells (Figure 3). Inflammatory cells, predominantly lymphocytes and plasma cells mixed with neutrophils, infiltrated the stroma. However, the glandular architecture was preserved, which was consistent with the normal findings observed on endoscopy. Finally, The multiplex PCR (The BioFire FilmArray Gastrointestinal Panel, BioFire Diagnostics, Salt Lake City, UT, USA; Table S1 lists the microorganisms that can be detected) tested positive for *Cryptosporidium*. *Cryptosporidium hominis* was subsequently identified in the stool specimens by 18S rRNA gene analysis.

Although we considered treatment with nitazoxanide, we started treatment with ART alone and monitored the clinical response. Diarrhea gradually improved to soft stools after ART initiation. He was discharged without additional therapy. During the follow‐up outpatient visit two months after starting ART, the patient had no further symptoms of diarrhea, and the CD4+ count had improved to 198/μL, and the viral load was not detectable.

## 4 Discussion

We reported a case of severe diarrhea caused by cryptosporidiosis in a person with HIV. Gram staining revealed white, unstained structures suggestive of protozoa (e.g., *Cryptosporidium*, *Cyclospora*). Based on Gram staining findings, we subsequently performed acid‐fast straining and multiplex PCR, which facilitated a rapid diagnosis and supported the early treatment strategy.

Traditionally, cryptosporidiosis has been diagnosed by microscopic identification of oocysts in the stool using acid‐fast staining or direct immunofluorescence staining. In many cases, definitive diagnosis of cryptosporidiosis based solely on endoscopic findings of inflammatory patterns in the stomach, duodenum, terminal ileum, and colon is challenging because of the absence of pathognomonic endoscopic features [6], although gastrointestinal endoscopy would be helpful to assist definitive diagnosis in combination with endoscopic biopsy for histopathology, as shown in this case. Thus, immunofluorescence microscopy is considered the gold standard for detecting *Cryptosporidium* in reference laboratories in the USA and Europe [7]. However, immunofluorescence microscopy is time‐consuming and requires a skilled microscope. Compared with immunofluorescent antibody staining, modified acid‐fast staining has a sensitivity of approximately 70% [8]. Gram staining of the stool specimen revealed structures with white halos suggestive of the presence of oocysts. This finding contributed to the subsequent diagnosis using the multiplex PCR. Gram staining is not a standard method for diagnosing cryptosporidiosis; however, it may be useful as a simple and convenient approach to detect *Cryptosporidium* oocysts screening, in addition to acid‐fast staining.

Cryptosporidiosis is not widely reported in Japan. Stool culture and amoebic antigen tests are often performed but are insufficient for diagnosing cryptosporidiosis. Although the multiplex PCR test is not yet common in Japan and is not covered by the Japanese public health insurance system (it is approved as an in vitro diagnostic agent), it may be a valuable tool for investigating diarrhea in immunocompromised patients, such as people with HIV.

The multiplex PCR has recently become available for the simultaneous detection and identification of common enteric protozoan parasites. For *Cryptosporidium*, the reported sensitivity and specificity of the assay were from 95% to 100% [9]. Despite its high diagnostic accuracy, a significant limitation of this technology is its high cost, which makes it unfeasible for routine clinical testing. A cost‐effective strategy could involve initial screening with Gram staining/acid‐fast staining, followed by multiplex PCR panel assays for stool samples that test positive by microscopy. Although cryptosporidiosis is notifiable under Japan's “Act on the Prevention of Infectious Diseases and Medical Care for Patients with Infectious Diseases” [10], only a few cases have been reported each year. Mild cases often remain untested, and the low sensitivity of conventional diagnostic methods may contribute to underdiagnosis. This case highlights the multiplex PCR. Although Gram staining/acid‐fast staining served as the trigger for testing in this instance, the multiplex PCR could facilitate diagnosis in people with HIV‐associated diarrhea without relying on Gram staining. Although cost‐effectiveness must be considered, the proactive use of multiplex PCR may help uncover the underreported cases of *Cryptosporidium*.

There is no definitive evidence supporting the efficacy of specific therapeutic agents in the management of cryptosporidiosis in immunocompromised individuals [11]. In people with advanced HIV, the cornerstone of treatment for cryptosporidiosis is ART with immune reconstitution [11]. While data on antimicrobial interventions remain inconclusive, some patients with severe or persistent symptoms that do not resolve following the initiation of ART may benefit from antiviral agents such as nitazoxanide and paromomycin [11, 12]. In the present case, we prioritized monitoring the response to ART initiation. As the individual's diarrhea resolved, the administration of antimicrobial agents was deemed unnecessary.

## 5 Conclusions

*Cryptosporidium* is widely recognized as a cause of diarrhea in people with HIV. However, its incidence has declined in recent years, and the prompt diagnosis remains challenging. In this case, Gram staining of the stool sample revealed structures with white halos, suggestive of oocysts. When such white, unstained structures are observed on Gram staining, particularly in people with HIV, a protozoal infection should be strongly suspected. Using a simple and rapid GI panel can lead to a more accurate diagnosis. This finding played a key role in confirming the diagnosis using the multiplex PCR supporting an early treatment strategy.

## Author Contributions

**Yudai Kono:** conceptualization, data curation, formal analysis, writing – original draft. **Mayu Arase:** conceptualization, data curation, writing – review and editing. **Kohei Ukai:** conceptualization, data curation, writing – review and editing. **Keiko Soneda:** conceptualization, data curation, writing – review and editing. **Shinya Yamamoto:** conceptualization, data curation, formal analysis, investigation, methodology, project administration, writing – review and editing. **Kazuhiko Ikeuchi:** writing – review and editing. **Eisuke Adachi:** conceptualization, project administration, supervision, writing – review and editing. **Yusuke Nomura:** data curation, investigation, writing – review and editing. **Yoshimi Higurashi:** data curation, investigation, writing – review and editing. **Shinji Izumiya:** data curation, investigation, writing – review and editing. **Kazuki Honjo:** data curation, investigation, writing – review and editing. **Yu Miyakawa:** data curation, methodology, writing – review and editing. **Sozaburo Ihara:** data curation, investigation, writing – review and editing. **Hiroyuki Abe:** data curation, investigation, writing – review and editing. **Mitsuhiro Fujishiro:** supervision, writing – review and editing. **Shu Okugawa:** supervision, writing – review and editing. **Takeya Tsutsumi:** supervision, writing – review and editing.

## Funding

The authors have nothing to report.

## Consent

Written informed consent was obtained from the patient for the publication of this case report (and any accompanying images) in accordance with the journal's patient consent policy.

## Conflicts of Interest

The authors declare no conflicts of interest.

## Supporting information


**Table S1:** The detectable microorganisms of multiplex PCR assay GI Pannel.

## Data Availability

No datasets were generated or analyzed during the study.
