# Supplementary material for: From Diagnostic Challenge to Clinical Success: Rapid Multiplex PCR Identification of Cryptosporidium in HIV‐Associated Refractory Diarrhea: A Case Report
Source: Clin Case Rep. 2026 Mar 13;14(3):e72301. doi: 10.1002/ccr3.72301 (PMC13093272; doi:10.1002/ccr3.72301)
Supplement: Supplementary file 1 — Table S1: The detectable microorganisms of multiplex PCR assay GI Pannel. [file CCR3-14-e72301-s001.docx]

| VIRUSES | BACTERIA | DIARRHEAGENIC  ESCHERICHIA COLI/SHIGELLA | PARASITES |
| --- | --- | --- | --- |
| *Adenovirus* F40/41 | *Campylobacter (C. jejuni / C. coli / C. upsaliensis)* | *Enteroaggregative E. coli* (EAEC) | *Cryptosporidium* |
| *Astrovirus* | *Clostridioides (Clostridium) difficile*(toxin A/B) | *Enteropathogenic E. coli* (EPEC) | *Cyclospora cayetanensis* |
| *Norovirus* GI/GII | *Plesiomonas shigelloides* | *Enterotoxigenic E. coli* (ETEC) *lt/st* | *Entamoeba histolytica* |
| *Rotavirus* A | *Salmonella* | *Shiga-like toxin-producing E. coli* (STEC) *stx1/stx2* | *Giardia lamblia* |
| *Sapovirus* (I, II, IV, and V) | *Yersinia enterocolitica* | *E. coli* O157 |  |
|  | *Vibrio (V. parahaemolyticus / V. vulnificus / V. cholerae)* | *Shigella/Enteroinvasive E. coli* (EIEC) |  |

Supplemental Table 1. The detectable microorganisms of multiplex PCR assay GI Pannel
